# Implementing the STEADY Wellness Program to Support Healthcare Workers throughout the COVID-19 Pandemic

**DOI:** 10.3390/healthcare10101830

**Published:** 2022-09-22

**Authors:** Melissa B. Korman, Rosalie Steinberg, Lina Gagliardi, Brenda Stewart, Carmen Llanos Acero, Joanne Davies, Robert Maunder, Thomas Walker, Tracey DasGupta, Lisa DiProspero, Mark Sinyor, Janet Ellis

**Affiliations:** 1 Sunnybrook Health Sciences Centre, Toronto, ON M4N 3M5, Canada; 2 University of Toronto, Toronto, ON M5T 2S8, Canada; 3 Sinai Health Systems, Toronto, ON M5G 1X5, Canada; 4 Ornge Air Medical Transport Operations, Toronto, Mississauga, ON L4W 5H8, Canada

**Keywords:** COVID-19, healthcare worker, implementation science, staff wellness, occupational stress, mental health

## Abstract

The COVID-19 pandemic has posed an ongoing threat to the mental wellbeing of countless individuals worldwide, with healthcare workers at particularly high risk. We developed the STEADY staff wellness program prior to the pandemic based on the available literature and input from stakeholders, guided by the Knowledge-to-Action (KTA) Implementation Science Framework. We quickly adapted the STEADY program for implementation in selected high-need units within Canada’s largest trauma hospital during the pandemic’s first wave. This brief report describes implementation of the STEADY program, retroactively applying the structure of the Knowledge-to-Action Implementation Science Framework to the practical steps taken. We identified the importance of more frequent, shorter contact with HCWs that occurred in-person, with an emphasis on peer support. A flexible approach with strong support from hospital leadership were key facilitators. Our findings suggest that a flexible approach to practical program implementation, theoretically underpinned in best-practices, can result in an acceptable program that promotes increased HCW wellbeing during a pandemic.

## 1. Introduction

Healthcare workers (HCWs) are at high risk of occupational stress injuries, with greater proportions of burnout, post-traumatic stress injury (PTSI) and suicide reported in this population than in the general public. PTSI and burnout severely impact work and home life for HCWs and harm their relationships with family and friends [1]. Additional stressors faced by HCWs during a pandemic further increased the risk of serious long-term psychological sequelae, including depression, anxiety, substance use, sleep deprivation, burnout, and PTSI [2,3,4,5,6,7,8,9,10,11,12], which continue to threaten the wellbeing of our healthcare workforce. Mental health and/or wellness interventions have been implemented to support HCWs throughout the COVID-19 pandemic [13,14,15,16]. As the COVID-19 pandemic required rapid implementation of such programming, with limited time for preparation, interventions were not necessarily evidence-based and were possibly implemented pre-maturely.

Premature implementation of a healthcare innovation can contribute to its failure or lack of future uptake, even if it is evidence-based, theory-driven, and/or efficacious in previous trials [17,18,19]. Thoughtful implementation strategies are necessary when setting up a program for success, especially when interventions aim to address issues as complex as occupational stress injury for HCWs who are routinely exposed to potentially traumatic events, who experience barriers to supportive care (e.g., stigma), and whose work environment is subject to time and resource-related challenges (further strained in pandemic situations). The chosen implementation approach can largely influence program successes or failures.

**Figure 1:**
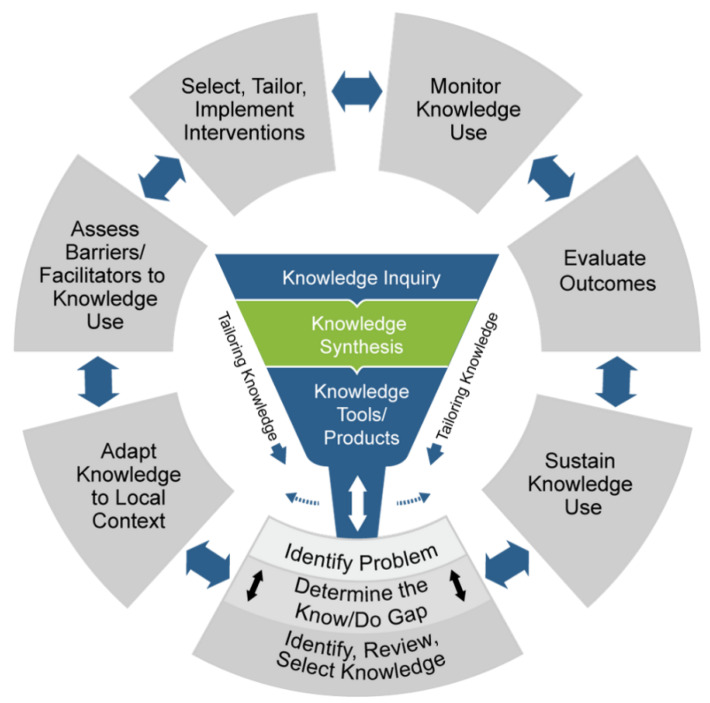
The Knowledge to Action Framework [20]. Re-printed with permission from the publisher (Wiley).

There is a growing body of literature regarding knowledge translation and implementation science, with many frameworks, theories and models created to guide researchers and policymakers through the development and implementation of healthcare innovations. The Knowledge-to-Action (KTA) Framework is widely recognized, employed, and promoted worldwide by organizations such as the Canadian Institutes for Health Research [20]. The KTA framework guides researchers and policymakers to translate evidence-based knowledge into action. In the current context, this could be leveraging the knowledge that effective post-traumatic stress injury prevention should include the means to increase social support [7,21] by implementing a buddy system to formalize mutual support between colleagues by assigning partners to check-in with one another, as has been recommended for HCWS based on previous outbreak situations [22]. There are two components of the KTA framework: (1) the Knowledge Creation Funnel; and (2) the Action Cycle, depicted as the centre funnel and outer circle of Figure 1, respectively [20].

Using a structured, iterative knowledge translation and implementation science approach, such as the KTA framework, encourages end-users (e.g., program facilitators) to engage in knowledge exchange with researchers/program developers. Such interactions, wherein end-users can share their experiences and perspectives of practical program implementation with researchers and policy makers, allows for mutual learning and iterative program adaptation to best suit the needs of the target population.

Gathering data regarding program implementation and effectiveness may not be well-planned during a crisis; many who implemented programming in response to COVID-related distress would not have evaluated outcomes. Prior research systematically evaluating the feasibility of implementing mental wellness programs in HCWs and the effectiveness of such programming is sparse, especially during disasters/pandemics. Most work has been conducted in ‘normal,’ non-pandemic times, or months-to-years following a disaster. HCW mental health programs developed or implemented using knowledge translation and implementation science frameworks is limited; the literature in the field is sparse.

Our team spent approximately three years prior to the COVID-19 pandemic developing the evidence-informed Social Support, Tracking Distress, Education And Discussion, communitY (STEADY) program, completing the Knowledge Creation component of the KTA framework [Figure 1]. Literature review on the topic, program development and description of the program are reported elsewhere [23]. Briefly, STEADY targets evidence-informed mediators of PTSI and resilience in first responder and healthcare worker populations (social support, earlier intervention for distress, substance use, stigma, and burnout). The program is composed of five key components: Peer Partnering, Wellness Assessments, Psychoeducation Workshops, Peer Support Discussions (general groups and critical incident stress debriefing) and Community-Building Activities. STEADY was designed to be flexibly adapted to any target population, in keeping with the KTA framework and considering select contextual factors outlined by the Consolidated Framework for Implementation Research (CFIR) [24].

Our team was involved in a response to staff wellness needs during the first wave of the COVID-19 pandemic at Sunnybrook Health Sciences Centre (SHSC), Canada’s largest trauma centre. This led to the implementation of the STEADY Program in select units at SHSC. Aspects of the KTA were considered throughout implementation, though the crisis-situation limited the capacity for a planned, structured approach. Our team implemented STEADY at SHSC as part of a Quality Improvement Project over one year of the COVID-19 pandemic. This narrative report describes the teams’ experience of practical implementation, describing steps taken in chronological order and retrospectively applying the KTA Cycle throughout, with embedded lessons learned and actionable recommendations.

## 2. Step 1: Response to Staff Wellness Needs

Following the COVID-19-related lockdown in Toronto, Canada, in March 2020, HCWs’ need for emotional support in coping with pandemic-related distress became evident to leadership. Our team was engaged in the response to wellness needs as part of the Hospital Emergency Operations Committee. We established a staff wellness working group which included psychiatrists, spiritual care providers, organizational development associates, occupational health and safety staff, hospital leadership, and members of the STEADY research team. We created a list of supportive resources (internal and external) to be sent to anyone requesting support, and a centralized email account to help staff access these resources. This included virtual one-on-one peer support session offerings with volunteers from the Department of Psychiatry. Volunteers provided times of the week that they were generally available, and we scheduled in those needing support.

## 3. Step 2: Planning STEADY Implementation (at the Organizational Level)

To add structure to the response, we applied the previously developed STEADY framework to support HCWs within the context of the pandemic. We had already completed the “identify the problem,” “determine the know/do gap” and “identify, review, and select knowledge” steps of the KTA Cycle while creating STEADY. Therefore, this project began at the “adapt knowledge to the local context” step of the action cycle. In order to do so, we needed to build a deeper understanding of staff needs in the context of the pandemic.

We previously amalgamated validated questionnaires of mediators of PTSI, anxiety, depression, and quality of work life (described in [23]) to make up the “Wellness Assessment” for the Tracking Distress (T) part of STEADY. Considering the many competing demands faced by HCWs and limited time available, we were asked to create a brief version to help elucidate organizational needs (which could then be used for the distress tracking component of STEADY). The brief assessment included demographic questions, (e.g., Position and Unit/Department) qualitative questions that varied from month-to-month (e.g., “How can Sunnybrook further support you in dealing with COVID and other current life stressors?”, “How has online schooling and the delay return of in-person classes affected you?”), as well as the Generalized Anxiety Disorder-2 [25], the Patient Health Questionnaire-2 [26], the Primary Care Post-traumatic Stress Disorder Screen for DSM-5 [27], and the Single Item Burnout Questionnaire [28].

LimeSurvey housed the Brief Wellness Assessment. Submissions were confidential, though participants could choose to provide their email address to receive tailored responses. A response algorithm was developed according to possible distress profiles; responses included a summary of results (e.g., what their scores revealed and whether their scores had improved or worsened since previous screening) and suggested resources. In the first month (April 2020), there were 947 submissions, with 282 respondents providing their email addresses.

The research team consulted with the working group and other organizational leaders to adapt the other elements of STEADY (peer partnering for social support, workshops and other psychoeducation resources, peer support sessions and critical incident stress debriefing for discussion of distress, and community-building activities) to the current context, considering pandemic-related restrictions (e.g., inability to have in-person gatherings). We presented our initial project plan [described below] to the Senior Leadership Team at SHSC and applied for research funding. A limited amount of organizational funding was allocated to our team to begin responding to high-needs units while we worked to secure grant funding to implement and evaluate the use of STEADY across a larger population, with Senior Leadership support.

### Initial Project Plan

This section outlines the initial plan created in March-April 2020. We proposed implementing the full STEADY program (as outlined in Table 1) in eight high-need units, the “STEADY units,” for six months. Units would be selected based on need, identified through Wellness Assessment data, absenteeism data, and through conversations with relevant leadership. We would meet with unit management to adapt each element of STEADY to the specific context, and then offer a variety of interventions to the group so that staff could select what they were most interested in.

Ethically and organizationally, we needed a strategy to respond to distress in “non-STEADY units.” Some resources would continue to be offered organization-wide, including the brief version of the Wellness Assessment being sent out monthly to continue monitoring wellbeing across the organization and identifying areas of distress or need. Findings from the Wellness Assessments were reported back in monthly working group meetings, and specific areas of need or requests that leadership might be able to address were fed back to organizational leaders.

## 4. Step 3: Planning STEADY Implementation (at the Unit Level)

This began as a quality improvement project rather than a research project (the Research Ethics Board at SHSC confirmed that ethics approval was not needed); the project underwent peer-review during the grant review process. Funding was acquired in May 2020 to implement STEADY in eight of the highest distress units at SHSC (according to Wellness Assessment data, leadership observation and sick time data) for a six month period. We selected five units at SHSC’s acute care site and the entire rehabilitation site (five additional inpatient units plus other departments) for program implementation.

Moving clockwise on the KTA action cycle, we “assessed barriers/facilitators to knowledge use” by meeting with managers and/or staff delegated by the managers of each target unit to gauge which elements of STEADY they believed would be most acceptable to their group, while gathering logistical details of potential barriers to implementation. Throughout this process, we considered constructs within the “inner setting” domain of the CFIR, including “culture” and “implementation climate.” We asked how open the group might be to different types of support, how and where the team preferred to gather for sessions, and for the manager/delegate’s opinions on our plans. Feedback was used to “select, tailor, and implement interventions,” by informing the implementation logistics, such as the best method of peer partnering, and realistic frequencies for offering peer support sessions and education workshops. Resulting plans for implementation looked slightly different between groups [Table 2].

Monthly team meetings were planned, as a way for the team to accomplish the “monitor knowledge use” step of the KTA cycle. We planned to log provision of partner assignments, surveys, workshops, and peer support groups, as well as number of attendees at each STEADY session, as a proxy for program feasibility and acceptability, and to “evaluate outcomes” (another step on the KTA Cycle).

## 5. Step 4: Implementing and Adapting STEADY

Six individuals (mental healthcare practitioners and/or peer support facilitators) acted as STEADY peer support facilitators at the time of initial program implementation, two others joined this initiative during the process. Two STEADY peer support facilitators co-led programming in each unit (maintaining consistent facilitators in each group, wherever possible). One facilitator, who also acted as the program manager [MBK], worked with all participating units.

We began programming with each unit as soon as preparatory meetings were completed for that group, due to the urgent nature of the pandemic. STEADY was introduced to each participating unit via an e-mail sent to the group by their patient care manager (using a template provided by the STEADY program manager) between May and October 2020. Introductory e-mails included a general introduction to the programming, an offer to pair up individuals with a partner (asking staff to respond with 3–5 names of individuals they would be comfortable partnering with, as a means of ‘opting-in’ to this component) and access to both the brief and extended versions of the wellness assessment via LimeSurvey. Research assistants checked LimeSurvey for submissions on each weekday and reviewed scores to determine whether an individual scored positively according to the evaluation criteria for each validated measure used. Research assistants sent a response to any individual that provided their e-mail address, according to their combination of scores. For example, if they scored negatively for all areas of interest, respondents were told that their scores indicate that they are coping well, they were reminded to practice self-care and provided general resources created for healthcare workers or for managing stress during the pandemic. Response templates can be accessed by contacting the corresponding author. Clinical STEADY team leaders were engaged whenever individuals included concerning comments or requested specific types of support, and when scores were consistently high.

Select facilitators [JE, MBK, RS] and research staff were involved in workshop material development. Topics were identified based on past work, challenges highlighted by the literature, themes that arose in conversation with participants, and suggestions from other facilitators, participating HCWs or leadership. Each topic was offered two or more times on each unit, to increase accessibility. The final list of workshop titles was as follows:

1. Introduction to Self-Care;
2. Introduction to Meditation and Mindfulness;
3. Mindfulness Practice (Continued);
4. Becoming a Reflective Practitioner: Personal Practice and Reflective Communication;
5. Having Difficult (Death and Dying) Conversations with Patients;
6. Understanding and Overcoming Burnout and Compassion Fatigue;
7. Grief and Bereavement as a Healthcare Provider;
8. Normal Stress Reactions versus Acute and Post-traumatic Stress Disorders;
9. Skills for Coping and Resilience-Building;
10. Managing Anger (in yourself and in patients);
11. Tackling COVID-19- Related Stress;
12. Coronasomnia: Sleep and Mental Health;
13. Moral Distress.

Participation was strictly voluntary; individuals could pick and choose elements of the program that they wished to participate in. Informal conversations between program facilitators (i.e., end-users), HCW attendees, unit leaders and STEADY Team members were crucial in accomplishing the “monitor knowledge use” step of the KTA cycle. Having one consistent facilitator who acted as the STEADY Program Manager across all units (working with varying co-facilitators in each group) was beneficial for knowledge synthesis and change management. Some changes were made to adapt to HCWs’ needs and preferences across multiple units, while other changes were unit-specific. For instance, one-hour education workshops were deemed unrealistic across all units since HCWs were unable to take this time away from their clinical duties, especially considering increased clinical demands created by the pandemic. We adapted the frequency and duration of these workshops from biweekly one-hour to weekly thirty-minute workshops. Other adaptations were made to increase uptake or respond to specific units’ needs (rather than in response to challenges) as we learned more about their staff, unique unit practices and cultures. For instance, the Acute Care Inpatient Unit described in Table 2 initially asked for education workshops as the only interactive programming facilitated by the STEADY team, as they thought peer support sessions would not be acceptable to the group. However, workshop facilitators identified a desire for peer support sessions. We adapted programming by offering weekly peer support sessions in addition to the weekly education workshops.

The “community” element of STEADY was difficult to implement consistently due to lack of structure. In some areas where leaders actively planned holiday events, we were welcomed to participate in or co-host these celebrations, thereby working with leaders to bolster group interaction and sense of community. In other areas, leaders attended education workshops to encourage staff attendance and model vulnerability. However, this element was not implemented in all STEADY units until we decided to create communal “Gratitude Trees” for each participating group, with manager consent. This idea arose from the monthly working group meetings. These meetings created the opportunity for interdepartmental conversations about the various wellness initiatives being run and unaddressed needs across the organization, in addition to discussing progress with STEADY.

### 5.1. Challenges Faced and Solutions Implemented

It quickly became clear that remote/virtual program implementation was not acceptable to most groups, with minimal or no uptake of remote workshops or peer support sessions. To address this, we received organizational approval for one facilitator to attend sessions in person, following infection prevention and control protocols, and “zoom in” the second facilitator. This hybrid approach allowed one facilitator to build relationships with individuals during casual conversations in their area, even if they had not yet attended programming. It helped to maintain physical distancing and to increase accessibility, as staff on target units could choose to attend from home on days off. The extra time needed to make these in-person changes, build trust with staff and gain traction on the units led to a project extension beyond the initial planned six months to approximately eight-to-12 months. Other challenges faced and solutions implemented are outlined in Table 3.

### 5.2. Non-STEADY Units

Specific protocols for responding to high-distress areas were developed in real-time. Where increased distress was identified by a unit manager or team member, the STEADY team met with leadership/management, wherever possible, to determine how to prioritize and target interventions for their staff. This often included a Group Wellness Assessment, using a peer support model to engage staff and normalize conversations about distress, validate their experiences, and identify issues. Further supports were suggested based on identified issues or reported desires. For example, where staff described experiencing burnout and the desire for more opportunities to connect as peers, we offered the workshop on “understanding and overcoming burnout and compassion fatigue” and created the opportunity for peer support discussions during these sessions. Other departments were engaged when concerns fell outside the scope STEADY. For instance, the Department of Organizational Development and Leadership was engaged for team-building interventions where team dynamic issues were described.

## 6. Step 5: Evaluating Program Outcomes

In the absence of other evaluative strategies to gather impact data from participants, we collected comments and feedback that were offered spontaneously over the course of programming. Feedback was virtually entirely positive; Table 4 includes examples. Requests for support were received from approximately twenty non-target units based on positive word-of-mouth, indicating program acceptability and success. Based on expressed benefit of programming and continued need, the Senior Leadership Team funded STEADY programming for seven months beyond the original planned timeline. Even after the project has ceased, our team continues to receive requests for support, and to work with the Organizational Wellbeing@Sunnybrook Committee to respond as appropriate.

## 7. Discussion

Program implementation required iterative bi-directional movement across four parts of the KTA Action Cycle: (1) Adapt Knowledge to Local Context, (2) Assess Barriers/Facilitators to Knowledge Use, (3) Select, Tailor, Implement Interventions, and (4) Monitor Knowledge Use. Feasibility and acceptability data are being analyzed, though analysis is complicated by the iterative adaptations of programming across the participating units over time. Though this may have been due, in part, to the responsive nature of program implementation and need for expediency to address growing staff distress, it is in keeping with the rationale for the bidirectional arrows on the KTA Action Cycle. If possible, future program implementation might benefit from a prolonged planning stage wherein a larger number of the target population are surveyed or consulted to identify barriers/facilitators to knowledge use and ways to tailor implementation (i.e., more time to assess barriers/facilitators to knowledge use, and to consider additional constructs from the CFIR). Nonetheless, future work should include extensive logs with findings from informal conversations regarding barriers/facilitators to knowledge use and ways to tailor interventions, dates that each adaptation was implemented, and observations of program facilitators/end-users related to these adaptations. This would allow teams to evaluate differing iterations of their program/intervention to better understand team needs and guide future improvements.

The largest initial barrier to program implementation experienced by facilitators was a lack of engagement in virtual sessions. Our team arranged with someone on-site to connect a communal computer to Zoom, or for a space where staff could go to connect to Zoom, making staff aware of this location through e-mail invitations for sessions. Rates of participation were relatively low, if any, but this increased slightly after having built relationships with staff who could then announce to the group that the sessions were happening (for instance, if the facilitator was ill and unable to come on-site, after we had conducted months of in-person programming). Based on the feedback received from participants, as well as the facilitators’ observations/ understanding, and in keeping with the literature, likely explanations for this include the following:

1. **Lack of reminders**: Frontline HCWs do not typically sit at a desk with an open calendar. The large number of competing clinical demands sometimes results in staff “catching up” on charting and entering data into systems at the end of the day, and they are likely to forget that sessions are happening at a specific time during busy shifts, especially if they have not logged into their emails to receive the reminders sent electronically;
2. **Limited capacity to connect to Zoom:** Prior to the pandemic there was no need for webcams and/or microphones on computers in patient care areas. Delays in installation during the pandemic limited the capacity for remote access for those working in patient care areas (compared to management or administrative staff);
3. **Lack of familiarity with facilitators**: Facilitators did not have the chance to build rapport with target groups before asking them to login to sessions and discuss personal topics. The lack of trust and comfort with facilitators may have prevented individuals from joining;
4. **Stigma:** Logging into a session is, in essence, asking for emotional support, and one might feel that this is “admitting weakness.” Staff spaces in patient care areas are communal, and one might not feel comfortable logging in on a shared computer with other individuals around;
5. **Frustration:** Some groups expressed frustration in hearing messages that “we are all in this together,” when they felt that they were risking their safety by coming in to work every day while others stayed at home. This led to individuals rejecting online programming, feeling there was no genuine sense of caring and understanding of their situation shown through this mode of intervention.

Having at least one facilitator on-site was effective in increasing program uptake. Other effective strategies included being flexible in coming to the staff and working around their schedules and identifying “Peer Champions” from the unit who could encourage spread awareness, remind their peers, and co-facilitate sessions.

Some units were more open to programming than others. The construct of “culture” as part of the “inner setting” domain of the CFIR was experienced by facilitators to be particularly important with this type of program. It was important to try to understand each unit’s individual culture and offer programming in ways that would be acceptable based on unit norms. We observed greater difficulty breaking into the culture on trauma units (i.e., Emergency and Critical Care). A facilitator of implementation in these, and other, units was the support and encouragement of local leadership. Facilitators felt that it was easier to build trust in a group when leaders welcomed us in, introduced us to the group, spoke positively to staff about our work, and modelled vulnerability or openness to having conversations related to emotional wellbeing.

Despite challenges faced, we slowly gained traction, earned their trust, and became “part of the team,” to the point that some staff would reach out to us if they were distressed on days that we were not coming to their unit. Additionally, individuals reported missing us on weeks we were not there, and an overwhelming number of additional requests were received from other teams based on positive word-of-mouth. To accommodate this, we began to offer a weekly organization-wide workshop series via Zoom.

Informal peer support sessions (conducted as “walkabouts” or “team station check-ins”) were particularly successful. As many staff had recognized us as peer support facilitators by the time these were implemented, they quickly opened-up when we came to them. The more intimate and informal setting seemed to help staff feel comfortable openly discussing their wellbeing.

Gratitude trees were implemented in response to the theme identified by STEADY facilitators of a lack of gratitude/appreciation felt by staff. This consisted of our team hanging a large cut-out of a tree on a wall in the unit and providing paper ‘leaves’ for staff to fill out, by writing down something that they were grateful for, and add to the trees. This ‘community-building activity’ was very successful on most units—we even saw patients and their family members adding leaves with messages of gratitude for staff. Many of these trees are still posted, continuously added to, and valued. Other groups continue to request gratitude trees in their areas or advice in making them.

We continue to receive an abundance of positive feedback. There was no natural stopping time, as stressors continuously arose with the ongoing pandemic (including new variants and increased acuity of non-COVID patients), adding to the pre-existing (non-pandemic related) heightened risk for adverse mental health outcomes in HCW. Staff also became accustomed to having our facilitators provide peer support and expressed disappointment and frustration when we reminded them that the initiative would be ending. As a result, organizational funding was granted to extend the project timeline twice, and we were asked to transition programming to the organizational Wellbeing Committee for long-term sustainability.

We plan to adapt STEADY to create a peer-led version (STEADY-P), with less external support. This would enable teams to “sustain knowledge use” in the long-term and increase capacity for rapid upscaling to offer more widespread support to HCWs and potentially other high-risk groups.

## 8. Conclusions and Implications for Future Work

STEADY implementation was continuously adapted over approximately sixteen months of the COVID-19 pandemic. Many lessons were learned as we moved from theoretical program planning to practical, real-world implementation. Many of these learnings may be applicable to implementation of other HCW mental wellness programs (beyond STEADY). We will now summarize key lessons that should be considered when conducting similar work:

- **Peer Champions:** Include and/or train staff from the target groups to help run programming, for program acceptability and long-term sustainability. External facilitators should be involved alongside Peer Champions to bring objectivity and encourage the sense of a formal peer support environment, rather than that of ‘friends talking.’ Though this is recommended, we should note that during times of staffing shortages and high patient volumes (like the conditions during the pandemic), it might not be possible to train Peers Champions. From our experience, having unit staff advocate for programming and ‘co-facilitate’ by modelling vulnerability and sharing their own experiences helps to increase unit buy-in;
- **Find allies in leadership**: Unit and organizational leaders played a crucial role in paving the way for STEADY to be implemented, and in facilitating program uptake. Leadership modelling their trust in program facilitators seemed to encourage frontline staff to trust in the facilitators. In non-crisis situations, groups should take the time to engage leadership and build relationships with management in any target area;
- **If you build it, they won’t necessarily come:** Whenever possible, teams should go to the target unit rather than expecting them to come to you, ideally directly to where staff are already working. This gives staff the opportunity to engage in an informal, unpressurized way, and helps to overcome practical, logistical challenges (e.g., staff cannot always leave the bedside) and cultural challenges (e.g., HCWs don’t often prioritize their own needs as they are trained to focus on caring for others);
- **Maintain a flexible program structure:** Structure is important to maintain fidelity and ensure that elements are not forgotten in busy work environments (especially in the chaos of a pandemic), but flexibility regarding both date/time and method of delivery have proven to be key to the feasibility and acceptability of programming;
- **Keep calm and carry on:** Gaining traction takes time, and even once staff have bought in there may be days where only one individual will engage in a session. It is important not to take this personally or jump to conclusions regarding staff not needing or wanting support. Over time, we gained traction, saw the remarkable value and meaning in the smaller group conversations, and heard from many that they felt supported just knowing we were there and in seeing us on the unit;
- **Consider whether and how to end:** We implemented STEADY to respond to COVID-19 related distress and did not consider that distress may be even higher after the pandemic. We found that COVID-19 magnified longstanding issues, and as staff experienced the effects of cumulative stress, distress grew, leaving staff needing more support (rather than less). Though, theoretically, using a peer support approach and training staff within the unit should be sustainable, creating this infrastructure amidst a pandemic/period of heightened stress and workload was particularly difficult. We did not find a “natural stopping point,” and relied on organizational resources to continue offering support to staff.

Based on feedback received and our observations over the course of programming, we conclude that this project was highly successful. We hope that reported lessons learned through the iterative process of program adaptation and actionable recommendations made will support others in similar endeavours.

## Figures and Tables

**Table 1:** Initial adaptation of STEADY elements planned for target “STEADY units”.

Element of STEADY	Initial Plan
Peer Partnering (for social support)	We planned to pair up dyads to act as “partners” or, where not feasible, encouraged staff to choose partners at each shift. Partners were encouraged to check in, provide mutual emotional support and encourage self-care, especially following a critical incident.
Wellness Assessments (for tracking and early identification of distress)	We proposed to offer both the “Brief Wellness Assessment” (described in the main text) and the “Extended Wellness Assessment,” i.e., the initial set of validated questionnaires amalgamated upon STEADY development, to the target units on a monthly basis over the height of the pandemic. Those who opted to complete the extended version could receive more personalized responses/suggestions. For example, those that reported difficulty sleeping could receive specific suggestions for improving quality of sleep.
Workshops and resources for psychoeducation	We planned workshops on a range of topics related to mental health, coping and resilience building. Some had been developed for the previous pilot project (e.g., Introduction to Self-Care and Becoming a Reflective Practitioner: Personal Practice and Reflective Communications), and new topics were to be added as appropriate. Frequency was to be determined based on the needs, interests and working environments of each target group. A STEADY webpage and online workshop modules were planned.
Peer Support Sessions and Critical Incident Stress Debriefing (for discussion)	We planned to facilitate virtual peer support sessions once or twice weekly per unit during the height of the pandemic. We partnered with Ornge Air Medical Transport Operations to train our team and other staff at SHSC in Critical Incident Stress Debriefing, who could then provide debriefings upon request. The goal of these debriefs would be to increase processing, communication and discussion, rather than for operational needs or causal analysis.
Community-Building Activities	As a STEADY webpage had not yet been developed, we worked with the Departments of Occupational Health, Organizational Development and Leadership, and Communications at SHSC to add to the resources available on the centralized “Be Well” webpage and increase accessibility of resources. We trained leaders to model vulnerability, de-stigmatize shared human distress and highlight the importance of self-care during Leadership Orientation Sessions. These sessions also covered principles of leadership in a disaster situation, ways to support teams through a crisis, an overview of the rationale for and evidence behind STEADY, ways to incorporate elements of STEADY into a team environment, reminders of hospital resources (e.g., the “Be Well” page), and introduction to peer support sessions and other resources accessible via the dedicated email. There were approximately 260 attendees across 4 Virtual sessions. Finally, we planned to invite HCWs’ family members to participate in STEADY, wherever possible and acceptable to staff, in the hopes that this would help family members understand the impact of occupational stressors and be able to support someone through them. We hoped that supporting family members would normalize conversations about distress and promote effective support at home, while allowing them to feel a sense of community.

**Table 2:** Examples of initial implementation plans for differing units determined by STEADY team in conjunction with target group leadership and relevant staff.

Site	Acute Care Inpatient Unit	Emergency Department	Outpatient Services Area	Rehab site
**Plan**	Peer partnering offered (individuals asked to send 3–5 names to the STEADY team of individuals with whom they would be comfortable partnering) Wellness Assessments emailed out monthly by STEADY team Team instructed to contact us for critical incident stress debriefing, as needed			
	No peer support sessions	Weekly peer support sessions	Weekly peer support sessions	Peer support sessions on inpatient units (rotating between units weekly) plus biweekly site-wide sessions
	Weekly 30-min education workshops (with opportunity for peer support)	Biweekly 1-h education workshops	Biweekly 1-h education workshops	Weekly 30-min site-wide workshops

**Table 3:** Lessons learned throughout STEADY Implementation: Challenges faced (in some or all target groups) and solutions implemented.

Challenge Identified.	Solutions Implemented/Lessons Learned
Gaining traction was particularly challenging in certain areas due to the group culture (i.e., stronger stigma against discussing distress with staff uncomfortable discussing personal needs or focusing on the *unit* rather than the *self*)	Trust was gained during consistent unit visits over time Peer Champions or group leaders were asked to model vulnerability, and facilitator self-disclosure was used strategically Not all conversations weree distress-related. Sometimes all staff needed was to talk about something light (like a current favourite show) to have a mental break from the work environment, or to connect with someone.
Individuals were wary of external personnel coming to discuss personal feelings and staff needs	Attendance improved significantly with Peer Champion co-facilitators (compared to sessions run by external STEADY facilitators only) Team Leader buy-in is very beneficial (where staff were comfortable with their leader) Assurances of confidentiality were made, and trust was built as staff saw there were no repercussions over time
Human Resources and pre-existing team dynamic issues raised in group discussions	Added to the shared group guidelines, or “Comfort Agreement,” that “naming and blaming” would not be permitted We liaised with Organizational Development to provide team building work where appropriate/needed Regular meetings with HR and Occupational Health to share systemic themes for action to be taken, where possible
Formal peer support groups and workshops held in a separate room and/or off-unit were not feasible (staff could not always get away from bedside, or would choose not to attend)	Uptake increased when we came to them vs. them coming to us: Walk-through check-ins conducted when staff could not leave the bedside, where facilitators did “laps” of the units, stopping to check in with staff individually or in small groups Where staff did not need to remain at bedside, informal sessions were offered at the central team station (staff may feel less vulnerable “casually” contributing to conversation rather than actively going to a session) Workshops conducted at central team station enabled staff to listen in while charting, etc. rather than feeling the need to stop other work to attend Timing of groups/workshops adjusted according to HCW schedules/requests (e.g., end of shift, lunch hour)
Timing is difficult to predict in the uncertain HCW work environment	Flexibility was important for our group; on days where units were particularly busy, we would reschedule last minute (i.e., upon arrival)

**Table 4:** Select messages of thanks and positive feedback received from STEADY participants.

Format	Message
Comments from Wellness Assessments	This program provided me much needed support, so I thank you for your dedication in employee services.
	STEADY is awesome and we love you guys. Thank you for all you do to help us.
Written Comments from Workshop Attendees	I am grateful for this session, being able to hear it and attend it. Thank you so much for all of your information and stories. It is nice to know that people are experiencing similar things and to hear about all the different strategies to practice self-care.
	Thank you for this session. It has been great to know that feelings of stress and compassion fatigue are experienced by many—I think being able to recognize these signs will be helpful for my own self, and for how I respond to others under this stress. The ideas and suggestions to improve wellbeing are wonderful.
Paraphrased Comment from Workshop Attendee	I hear your voice in my head almost daily telling me to balance to activities that nourish and deplete me and do things that I am inclined not to because it will be nourishing.
Email messages	I wanted to thank you for your wonderful program being offered to our clinical staff during this challenging times. Many of our nurses, clinicians provided a very positive feedback and would appreciate for a similar support in the future.
	Many thanks to you both and your teams for making the STEADY resource available for staff during this pandemic
	Thanks for coming by [our area] yesterday! It was very uplifting!!!
	I would like to express much appreciation and gratitude for all of the care and commitment to staff wellness the STEADY team has been diligently doing all these months.

## Data Availability

Not Applicable.
